# Assessment of Treatment Outcome and Its Associated Factors among Adult Epileptic Patients in Public Hospitals in the Southern Ethiopia: A Multi-center Cross-sectional Study

**DOI:** 10.4314/ejhs.v33i2.18

**Published:** 2023-03

**Authors:** Muluken Ahmed, Mohammed Nasir, Solomon Yalew, Firdawek Getahun, Fitsum Getahun

**Affiliations:** 1 Pediatrics department, College of Medicine and Health Sciences, Arba Minch University, Arba Minch, Ethiopia; 2 Pediatrics department, College of Medicine and Health Sciences, Hawassa University, Hawassa, Ethiopia; 3 School of public health, College of Medicine and Health Sciences, Arba Minch University, Arba Minch, Ethiopia; 4 Internal medicine department, College of Medicine and Health Sciences, Arba Minch University, Arba Minch, Ethiopia

**Keywords:** Epilepsy, seizure, anti-epileptic drugs, good control, poor control, poor adherence, good adherence

## Abstract

**Background:**

Epilepsy accounts for 0.5 % of the world's disease burden. Around 80 % of these are living in low and middle-income countries. In Ethiopia, the prevalence is 0.6 to 5 per 1000 population. There is a little study in our study area on the treatment and predictors of response of adult epilepsy. The purpose of this study was to determine the treatment outcome and its associated factors among adult epileptic patients in public hospitals in southern Ethiopia.

**Methods:**

Multi-centered, Hospital-based cross-sectional study was conducted from October 2021 - august 2022. Data were collected by face-to-face interviews and record review. Data was analysed using SPSS. The bivariate and multivariable logistic regression analyses have been performed between the dependent and the independent variables.

**Result:**

Of the total 422 participants, 55.9 % were males and 62.6% were below 30 years of age. The most common type of seizure was a generalized tonic-clonic seizure. Most (87.9 %) were treated by immunotherapy. Phenobarbitone is most common medication (77.5). One-quarter reported adverse effects of medication. The majority (78%) had good control (seizure free for at least one year) and 22% had poor control. Poor medication adherence (AOR=4.03) and shorter duration of seizure before treatment (AOR=4.233) were associated with poor control.

**Conclusion:**

A significant number of patients had poor control of seizures. Early identification of issues on medication adherence and early initiation of treatment will improve treatment outcome.

## Introduction

Epilepsy is a two or more unprovoked seizure that occurs due to electrical abnormality in the brain (1) Around 50 million people worldwide have epilepsy. Epilepsy accounts for a significant proportion of the world's disease burden. It accounts for 0.5% of the global burden of disease. It affects 3–4 % of people in developed countries and the figure may be higher in countries with low socioeconomic status. (2,3). Its prevalence in African countries ranges from 5.1/1,000 to 58/1,000. In Ethiopia, the prevalence of epilepsy is 0.6 to 5 per 1000 population. The highest age-specific incidence occurred in the youngest age groups (0–9 years); the next highest was in the group aged 10–19 years (4,5).

The international league against epilepsy (ILAE) 2017 version classifies epilepsy in three levels, first by seizure type, then by epilepsy type, and finally by etiology (6).

Based on the cause, there are two types of epilepsy; if there is identified cause it is called symptomatic otherwise it is idiopathic. The causes of symptomatic epilepsy could be stroke; hippocampal sclerosis, infections, and trauma. Focal seizure is more common than generalized seizure (7).

Although there are different modalities of treatment for epilepsy like antiepileptic medications, surgery, devices, and the ketogenic diet, most patients can be treated with antiepileptic medications. Indications of antiepileptic drug (AED) initiation are identified predisposition to recurrences like two unprovoked seizures occurring more than 24 hours apart, epileptiform abnormalities on electroencephalography, abnormal brain imaging, nocturnal seizures, or an epileptic syndrome (8).

The main goal of antiepileptic drugs is to control the seizures by minimizing the side effects of the drugs. So it should take into consideration of age, sex, type of seizure, concomitant medical illness, use of other medications, lifestyle and patient preference, and adverse effects. The first attempt to control the seizure must be with monotherapy. Polytherapy can be tried if there is difficulty in controlling the seizure with single AEDs. Older AEDs have a comparable seizure control rate, while newer AEDs are chosen for their better side effect profile. Older AEDs are frequently used in Sub-Saharan African countries for their long-term experience, lower cost, and known efficacy (8–12).

In the public sector with less than 50% availability of generic antiepileptic drugs only one-third of those people who had epilepsy get treatment(1). Not all epileptic patients have good control with antiepileptic drugs. Of those halves of patients can have controlled seizures with a low or moderate dose of single AEDs. Around 15% of patients may have poorly controlled seizures with two antiepileptic medications and be labeled as having drug-resistant epilepsy. In Finland, at the end of the 40-year median follow-up, since their first seizure before the age of 16 years, 95 (93%) of 101 patients had entered one or more one-year remissions (1YR). In contrast, 7% of patients never experienced any 1YR and their epilepsy was considered drug-resistant. Having weekly seizures during the first year of treatment and having weekly seizures before treatment are the factors associated with adverse outcomes (13).

In one study assessing a treatment outcome response pattern for AEDs, four patterns of outcome were identified. Patterns were early and sustained response, delayed and sustained response, fluctuating result, and no response. The order of the response was 37% had early and sustained response (seizure free for 1 year), 11% had delayed and sustained response and 16% of patients showed fluctuating results between periods of seizure freedom and relapse. Seizure freedom was more common in patients who were taking 1 than more than 1 AEDs, and 1 versus 3 AEDs. There was also a difference in the outcome of patients with symptomatic versus idiopathic seizures (9,14).

Research done in Nigeria in 1017 showed the use of polytherapy accounted for 14.46% of all treatment. The most common AED to be prescribed for the patient was Sodium valproate 46.31% of the prescriptions, followed by carbamazepine 18.41% and third was levetiracetam which accounted for 9.47% (12).

A study done on 780 patients with follow-up period of 10 years showed that predictors of pharmaco-resistance for AEDs were family history of epilepsy, previous febrile seizures, traumatic brain injury as cause of the epilepsy, intermittent recreational drug use, and prior or current psychiatric comorbidity, particularly depression (6,15,16).

Ethiopian studies done in different regions of the country tried to see a pattern of epilepsy and its treatment outcome. The generalized tonic-clonic seizure was the commonest type of seizure. Monotherapy was used in most patients and phenobarbitone was the preferred drug monotherapy. Good control of seizure is 40 to 80 %. A frequent number of seizures before starting treatment and poor adherence to medications were the two common identified factors (17,20).

Depression and anxiety are also common (33 %) in the population with epilepsy. They have also 22 % more likely of committing suicide compared to general population (13). Other problems faced by people with epilepsy are stigma and discrimination. The stigma of the disease can discourage people from seeking treatment (21).

Because the impact of epilepsy involves the individual and family and indirectly the community, a study showing the treatment response and factors which affect the response is very important. Even though some studies are done in Ethiopia, there is no such study that involves many centers. Furthermore, to the best of our knowledge, no research has been conducted on the outcome of adult epilepsy and its associated factors in our study areas. As a result, the goal of this study was to determine the response to treatment of epilepsy in adults who have follow up at public hospitals in southern Ethiopia and identify factors associated with it.

## Method and Materials

**Study area and period**: The study was conducted in four hospitals in the Gamo Zone, Southern Nations, Nationalities, and Peoples' Region from October 2021 -August 2022. Gamo Zone is named for the Gamo people whose homelands lie in this zone. The administrative center of Gamo is Arba Minch town. Arba Minch town is located 505 km southwest of Addis Ababa, the capital city of Ethiopia. According to data from respective zonal offices, the estimated total population of Gamo is 1,544,756. In the Gamo zone, there are 18 Woredas, 56 Health centers, and 4 hospitals. Four hospitals (Arba Minch General hospital, Chencha Primary hospital, Kamba Primary hospital and Gresse primary hospital) were included in this study. Total 610 adult epileptic patients had follow up at these hospitals.

**Study design**: We conducted multi-centered, Hospital-based cross-sectional study in Southern Ethiopia public hospitals.

**Study population**: All adult epileptic patients who attended 4 hospitals in Gamo Zone, southern nation, were considered the study population. Epileptic patients aged ≥18 years and who had a minimum follow-up of 12 months were included. Those epileptic patients whose medical record/chart with incomplete data, unstable/aggressive patients, critically ill patients and those who have less than 12 months follow-up were excluded.

**Sample size determination**: The minimum sample size for this study was calculated using single population proportion with the following assumptions: 95% confidence level, 4% margin of error, 46.6% proportion of adults with good control from one research done in Ethiopia (22). The calculated sample size will be 302. By adding none response rate 10%, the minimum sample size for the study will be 332.

For the second objective, we calculate sample size using double proportion formula and calculated using Stata Version 14 statistical software. For sample size estimation, number of seizure before treatment and adherence to antiepileptic drugs are considered as major predictors of uncontrolled seizure. For number of seizure before treatment, we took CI=95 %, POWER=80 %, % of outcome among unexposed=20 %, OR= 1.98 and calculated sample size will be 319 (23). For second factor, adherence to medication, we took CI=95%, power=80 %, % of outcome among unexposed= 59.3, OR=2.1, and calculated sample size will be 422 (19). Based on the above formulae, sample size computed for the second objective is the larger and is taken for sample size estimate for this study, 422.

Then, the total sample size will be proportionally allocated for each hospital based on the number of patients' load. Accordingly: Arba Minch general hospital =300, Chencha primary hospital=45, Kamba primary hospital=35, Geresse Primary Hospital = 42.

**Data collection tool**: Data was collected by using a structured questionnaire and data abstraction sheet prepared by English language then translated to Amharic and gamocho language. The tool was designed by reviewing different literatures. The tools contain 4 parts: Socio-demographic data, medical history, treatment history, and medication adherence. A face-to-face interview and record review have been employed to collect data from study subjects. Data was collected and supervised by trained and experienced healthcare providers and principal/co-investigators. Two days of training were given both for data collectors and supervisors. The training focused on the purposes of the study, the content of the tool, the data collection technique, ethical issues, and the roles and responsibilities of data collectors and supervisors.

**Study variables**: The outcome variable for this study was the treatment outcome of adult epileptic patients. Treatment outcome could be a good outcome if no seizures in the last year or a poor outcome if there had been a seizure in the last year.

The independent variables were socio-demographic (Age, sex, marital status, educational level, residence, occupation,..), type of epilepsy, type of medication(polytherapy versus monotherapy), age of seizure onset, duration on treatment, type of seizure at onset, adherence to treatment, frequency of seizure before initiation of therapy and comorbidity.

**The following Terms and Operational:**

**Definitions are used**

**Epilepsy** is considered to be present when >1 unprovoked seizure occurs in a time frame of >24 hours, or one unprovoked seizure and a probability of further seizures to the general recurrence risk after two unprovoked seizures, occurring over the next 10 years or diagnosis of the epileptic syndrome.

**Controlled epilepsy** refers to seizure free for at least twelve consecutive months after antiepileptic therapy.

**Drug resistance epilepsy**: Patients whose seizures do not successfully respond to antiepileptic drug (AED) (two tolerated, appropriately chosen, and used AEDs) therapy are considered to have drug-resistant epilepsy (DRE).

**Status epilepticus** is seizure lasting greater than 5 minutes with no interruption or more than 1 seizure with no regaining of consciousness in between.

**Polytherapy** refers to the use of more than one AEDs for controlling seizure.

**Adherence**: The extent to which individuals take their medications as prescribed with respect to dosage and dosage interval

**Good adherence** is considered if the patient took equal or more than 90 % of his/her monthly medication.

**Poor adherence** is considered if the patient took less than 90 % of his/her monthly medication

**Data analysis**: The collected data were checked manually for completeness. Data were coded and entered into Epi-data software version 4.4.2.1 and then exported to the SPSS statistical package for analysis. Descriptive statistics were done and summarized by mean and standard deviation and presented in the form of tables and figures. Bivariate logistic regression analysis was performed between the dependent and each of the independent variables. Variables having a p-value of <0.25 in bivariate logistic regression was a potential candidate for multivariate logistic regression analysis to control confounders in regression models. Variables having a p-value of less than 0.05 in the multivariable logistic regression model were considered as statistically significant. Model fitness is checked by the Hosmer and Lemeshow Goodness of fit test. The strength of association between the outcome variable and independent variables is reported by using the adjusted odds ratio with 95% CI.

**Ethical considerations**: Before data collection, ethical clearance was obtained from the Institutional Research Ethics review board (IRB) of Arba Minch University, College of medicine and health sciences (IRB/1007/2021). Letter of the permission was obtained from respective bodies. Participation in the study was voluntary and the purpose of the study was explained to participants before conducting an interview. Written consent was obtained from study participants by attaching a statement of consent to each questionnaire. However, the identification of the participants was not recorded anywhere on the questionnaire, and confidentiality was assured by analyzing the data in aggregate. To protect the study participants and data collectors from the pandemic personal protective equipment was provided during the interview for the study participants and data collectors.

## Results

**Socio-demographic features of participants**: Among the participants, 236(55.9%) were male. Most of them were below the age of 30 yrs which occupy 62.9%. Those above 60 yrs of age occupied 3.3% of cases. More than half of the participants (55.5%) were single, and 39.3% were married. Regarding educational background, 74.2% respondents were able to at least read and write. Among the participants, 52.8 % were living in urban areas (Table 1).

**Table 1:** Socio-demographic features of study participants, 2021–2022

Variables	Category	Frequency (%)
Sex	Male	236 (55.9)
	Female	186 (44.1)
Age	≤ 30	264 (62.6)
	31–45	113 (26.8)
	45–60	31 (7.3)
	>60	14 (3.3)
Marital	Married	166 (39.3)
status	single	234 (55.5)
	Divorced	7 (1.7)
	Widowed	15 (3.6)
Educational	Illiterate	109 (25.8)
level	Can read and write	52 (12.3)
	Primary school	84 (19.9)
	attended	
	Secondary school	78 (18.5)
	attended	
	Diploma completed	58 (13.7)
	Degree completed	41 (9.7)
Monthly	<1000	240 (56.9)
income in	1000–2000	77 (18.2)
birr	2001–3000	41 (9.7)
	3001–4000	19 (4.5)
	4001–5000	21 (5.0)
	>5000	24 (5.7)
Occupation	Government employee	69 (16.4)
	NGO employee	47 (11.1)
	Merchant	9 (2.1)
	Farmer	103 (24.4)
	House wife	37 (8.8)
	Daily laborer	44 (10.4)
	Others	113 (26.8)
Residency	urban	223 (52.8)
	Rural	199 (47.2)

**Clinical characteristics of study participants**: The commonest seizure type patients had was a generalized tonic-clonic seizure (83.4%) and it is followed by an atonic seizure (6.2%). Two third of the case had seizures less than one year before the start of treatment (67.5%) whereas 12.3 % had a seizure for more than five years. Only 57 (13.5%) of the 422 respondents had EEG examination done. Fifty-nine cases were having comorbid conditions with their epilepsy. Psychiatric disorders (47.5 %) and hypertension (25.4%) are the two dominant comorbid illnesses. Of 422, 68 (16.1 %) had a history of head injury. When we see the number of attacks before the initiation of medication, 85.2 % of the patients had less than four attacks weekly. They mentioned triggering factor in 73.2% and emotional stress and missed medication in 39.2% and 38.8 %respectively (Table 2).

**Table 2:** Clinical characteristics of study participants, 2021–2022

Variables	Category	Frequency (%)
Type of seizure	GTC*	352 (83.4)
	Atonic	26 (6.2)
	Myoclonic	8 (1.9)
	Focal seizure	21 (5.0)
	Other	15 (3.6)
Duration of seizure before treatment	Under 1 year	285 (67.5)
	1–2 year	34 (8.1)
	2–5 years	51 (12.1)
	Above 5 years	52 (12.3)
EEG done	Yes	57 (13.5)
	No	365 (86.5)
History of head injury	Yes	68 (16.1)
	No	354 (83.9)
Time of head injury	Before onset of seizure	37 (54.4)
	After onset of seizure	31 (45.6)
Comorbid conditions	Yes	59 (14.0)
	No	363 (86.0)
Type of comorbid conditions	Psychiatric disorder	28 (47.5)
	Hypertension	15 (25.4)
	Diabetes melitus	9 (15.3)
	Heart failure	2 (3.4)
Trigger factor for the seizure	Yes	223 (73.2)
	No	199 (26.8)
Type of trigger	Emotional stress	121 (39.2)
	Missing medication	120 (38.8)
	Sleep deprivation	43 (13.9)
	Alcohol ingestion	17 (5.5)
	Khat and stimulant use	8 (2.6)

* GTC- generalized tonic clonic

**Figure 1:**
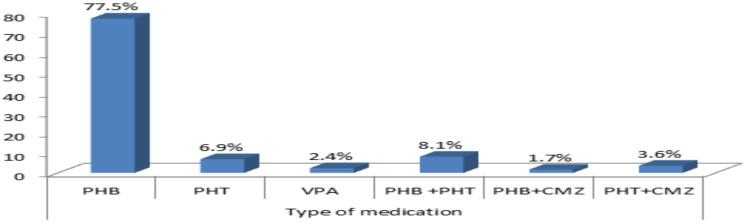
Type of Antiepileptic drugs used among patients with epilepsy attending the four hospitals Gamo zone, south Ethiopia, 2021

**Pattern of antiepileptic medication use among the study participants**: Single medication was used in 87.9 % of patients. Phenobarbitone (77.5%) was the most frequently prescribed AED among the study participants either as monotherapy or combination therapy followed by phenytoin (6.9%), and valproic acid (3.6%). Phenobarbitone (PHB) with phenytoin (PHT) was the most frequently used dual therapy regimen regarding AEDs use based on seizure type (Figure 1).

**Figure 2:**
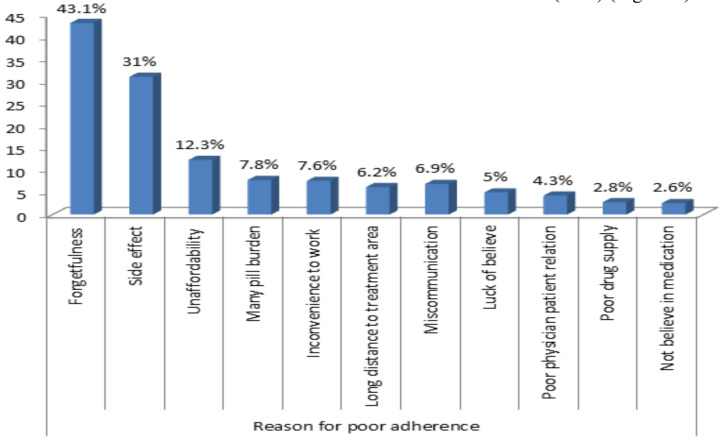
Reason for poor adherence among patients with epilepsy attending the four hospitals gamo zone, south Ethiopia, 2021.

Around one-quarter of the patients reported medication adverse effects. Sedation, fatigue, and confusion were the top three ADRs encountered reported by 41.2%, 19.8%, and 15.3% respectively. Regarding medication adherence, 72.7 % and 27.2 % had poor and good adherence respectively. Forgetfulness (43.1%) was identified as the most common reason for AED non adherence followed by side effects of medications (31%) (Figure 2).

**Treatment outcome and Factors associated with treatment outcome of participants**: Regard the outcome, 329 (78%) of the study participants have good outcome which is defined as no seizure for at least twelve months. In the bivariable logistic regression analysis, nine explanatory variables which had an association with treatment outcome and had p-value ≤ 0.25 were assessed for the final multivariable log regression model analysis. After multivariate logistic regression analysis was done, adherence to medication and duration of seizure before treatment are predictors of treatment outcome. Low medication adherence is associated with poor outcome (AOR=4.03, 95% CI=1.176 to 13.862, P=0.027) also short duration of seizure before treatment with AOR=4.233, 95% CI2.130 to 8.410, P=0.00 The odds of poor treatment outcome was 4.03 times higher in patients with low medication adherence compared to those patients with high medication adherence. The odd of poor treatment outcome was 4.44 times higher in patients with less than five years of diagnosis before treatment than greater than or equal to 5 years of diagnosis. In our study gender, age of patients, type and number of medications, and comorbid conditions are not associated with treatment outcome (Table 3).

**Table 3:** Treatment outcome and associated factors, 2021–2022

Variables	Seizure control		COR^a^ [95% CI^b^]	P- value	AOR^c^ [95% CI]	P- value

	Good, N (%)	Poor, N (%)				
**Sex**						
Male	178 (42.22)	58 (13.74)	1.41(0.88,2.25)	0.022	1.31(0.74,2.30)	0.626
Female	151 (35.78)	35 (8.29)	1		1	
**Marital status**						
Married	135 (32.0)	31 (7.35)	2.29(0.51,10.34)	0.031	1.69(0.27,10.68)	0.847
Single	174 (41.23)	60 (14.22)	3.45(0.78,15.19)		2.05(0.34,12.57)	
Divorced	20(4.74)	2(0.47)	1		1	
**Comorbidity**						
Yes	51 (12.09)	8 (1.90)	0.513(0.23,1.13)	0.018	0.57(0.22,1.50)	0.074
No	278 (65.88)	85 (20.14)	1		1	
**Polytheraphy with AEDs**						
Yes	54 (12.80)	10 (2.37)	0.61(0.29,1.26)	0.008	1.23(0.49,3.05)	0.212
No	275 (65.17)	83 (19.67)	1		1	
**Frequency of symptom**						
<4/wk	276 (65.40)	84 (19.90)	1.79(0.85,3.79)	0.005	1.58(0.66,3.79)	0.086
>4/wk	53 (12.56)	9 (2.13)	1		1	
**Medication adherence**						
Poor	234 (55.45)	73 (17.30)	2.12(0.80,5.62)	0.005	4.85(1.49,15.83)*	0.027
Good	95(22.51)	20 (4.72)	1	1	
**Duration of Disease before treatment**						
< 5 years	145 (34.36)	79 (18.72)	7.16(3.89,13.16)	0.010	4.44(2.29,8.59) *	0.00
≥ 5 years	184 (43.60)	14 (3.32)	1		1	
**Hx of head injury**						
Yes	59 (13.98)	10 (23.70)	0.56(0.27,1.15)	0.011	0.63(0.27,1.49)	0.411
No	270 (63.98)	83 (19.67)	1		1	

a COR: Crude odds ration

b CI: Confidence interval

c AOR: Adjusted odds ration

* Significant at P-value < 0.05

## Discussion

GTCS was the most common type of epilepsy identified from 83.4% of patients. (19, 24) But this figure is lower than studies from Gondar, Hawassa, Addis Ababa, and Ambo (17,18,23,24). The reason for a higher report of GTC could be most patients with focal and other forms of epilepsy have less severe symptoms to seek treatment. This study revealed that most patients are diagnosed solely with clinical history and neurologic examination without EEG findings (86.5%).

Regarding the type of treatment used, monotherapy was the most common regimen utilized (87.9%), which is higher than the findings reported from Mekelle (48.5), Addis Ababa (58.8), Hawassa (72%), India (62%) and, Taiwan (71%) studies (17,22,24,25). Of this Phenobarbitone was the most frequently prescribed AEDs among the study participants both as monotherapy and combination therapy followed by phenytoin and valproic acid. This finding is in line with studies done in Ethiopia (17,19,23,24). Contrary to this finding, Sodium valproate, phenytoin, and carbamazepine are the most frequently ordered medication in Nigeria, India, and Taiwan respectively (12,25,26). The most commonly used AEDs as polytherapy was the combination of phenobarbitone with phenytoin (8.1%) followed by the combination of phenytoin and carbamazepine (3.6%).

In approximately more than two third of our participants, seizure episode was eliminated with optimal AED medication (78%), and the remaining 22% of patients experience symptoms despite the use of AEDs. From this study, a higher number of participants had good treatment outcomes compared to other studies done in Ethiopia (17, 19, 23, 24). India and Scotland's studies also showed lower treatment response rates compared to our result (9,27) (Figue 3).

**Figure 3:**
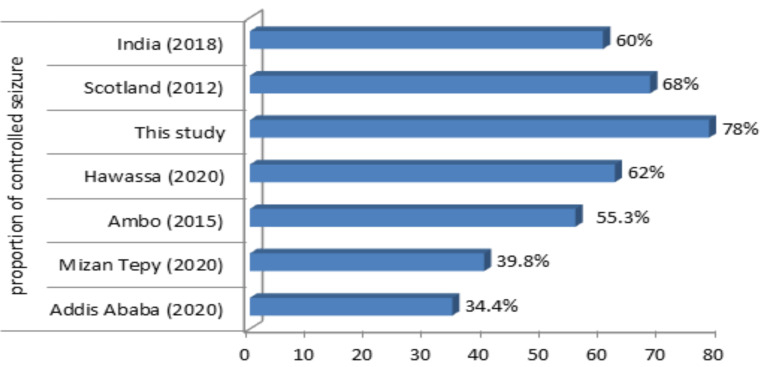
Comparison of good treatment response between other studies and this study

In the present study, around one-third of cases (31%) had developed AEDs related adverse effects. This is lower than the studies done in Mizan Tepi and Addis Ababa where 72.7% and 49.5% of patients experienced AEDs-related adverse effects, respectively (17,19). This difference could be due to the fact that most of the patients with GTCS in this study were on appropriate monotherapy (PHB). The second reason may be that in the current study the utilization of polytherapy as add on the initial regimen or polytherapy irrespective of AEDs, are potentially low (17% and 12 % respectively) which decreases the risk of drug-to-drug interaction which in turn reduces the risk of AEDs related adverse effects.

The result of this study also showed that poor medication adherence was an independent predictor of uncontrolled seizures. This is in line with the studies done in Gondar and Mekelle, Ethiopia (18,22). In this study, the most common reason for non-adherence was forgetfulness (43.1%).

The other predictor of treatment outcome was the duration of the disease before treatment. Duration of disease less than five years before initiation of treatment associated with poor outcome. This may be due to those who have severe and symptomatic seizure get treatment earlier than milder symptoms which can be controlled easily. This is against to the study done at Hawassa for which patients who are taking for less than one year have good outcomes this could be possibly due to high medication adherence and decrease risk of AED-related adverse effects (24).

The main limitation of this study was its cross sectional nature. There may be difficulty of recall issue. Other limitations are unavailability of imaging and EEG investigation to identify the cause for epilepsy and classify seizure. Only few cheap old antiepileptic drugs are available which make it difficult to compare other studies which participants uses newer with good adverse effect profiles.
